# An Uncommon Case of Partial Airway Obstruction due to Lingual Tonsillar Hypertrophy

**DOI:** 10.7759/cureus.8309

**Published:** 2020-05-27

**Authors:** Aveek Mukherjee, Raisa Ghosh, Anil Anandam

**Affiliations:** 1 Internal Medicine, Rutgers Robert Wood Johnson Medical School/Saint Peter's University Hospital, New Brunswick, USA; 2 Internal Medicine, Saint Peter's University Hospital, New Brunswick, USA

**Keywords:** lingual tonsil, tonsillar hypertrophy, airway obstruction, corticosteroid, hypoxia, waldeyer's ring, tonsil, difficult airway management, osa, mucosa-associated lymphoid tissue

## Abstract

Obstruction of the airway is a medical emergency. If it is not treated immediately, rapid and potentially life-threatening hypoxia develops. A 70-year-old woman with a history of hypertension and palatine tonsillectomy presented to our tertiary care hospital with dysphagia, odynophagia, muffled voice, and neck swelling of a one-week duration. She also complained of associated shortness of breath that began two days prior to hospital admittance. Bedside laryngoscopy revealed an enlarged base of the tongue and laryngeal edema, resulting in partial airway obstruction. A CT scan of the soft tissue of the neck revealed that lingual tonsillar hypertrophy (LTH) was the cause of the partial airway obstruction. While being closely monitored, the patient was treated with intravenous corticosteroids and antibiotics. Serial laryngoscopies were performed to track the resolution of the airway obstruction. Her hospital course remained uneventful, and the patient was discharged after four days. Though rare, LTH has a strong propensity to cause airway compromise, and it must be treated at once.

## Introduction

On the way to the larynx, air first passes through the oropharynx and nasopharynx. Blockages of these regions may result in airway obstruction and subsequent rapid and potentially life-threatening hypoxia, a medical emergency. Rarely encountered in routine medical practice, airway obstruction more commonly occurs during the administration of general anesthesia [1]. Here, we present the case of a 70-year-old woman who was admitted to our tertiary care hospital with dysphagia, odynophagia, hoarseness, and shortness of breath and was eventually diagnosed with lingual tonsillar hypertrophy (LTH), an uncommon cause of airway obstruction.

## Case presentation

A 70-year-old woman with a history of hypertension and palatine tonsillectomy presented to our tertiary care hospital with progressively worsening dysphagia, odynophagia, muffled voice, and neck swelling of a one-week duration. Shortness of breath began two days prior to hospital presentation and was exacerbated by lying supine. Bilateral neck swellings were located below the midsections of the mandibular rami. The patient did not report fever, sore throat, cough, or congestion, but did experience occasional chills. No allergies or new medications were documented. The patient was referred to our facility from her otorhinolaryngologist, who had performed an indirect laryngoscopy and was concerned about the patency of the airway.

**Figure 1:**
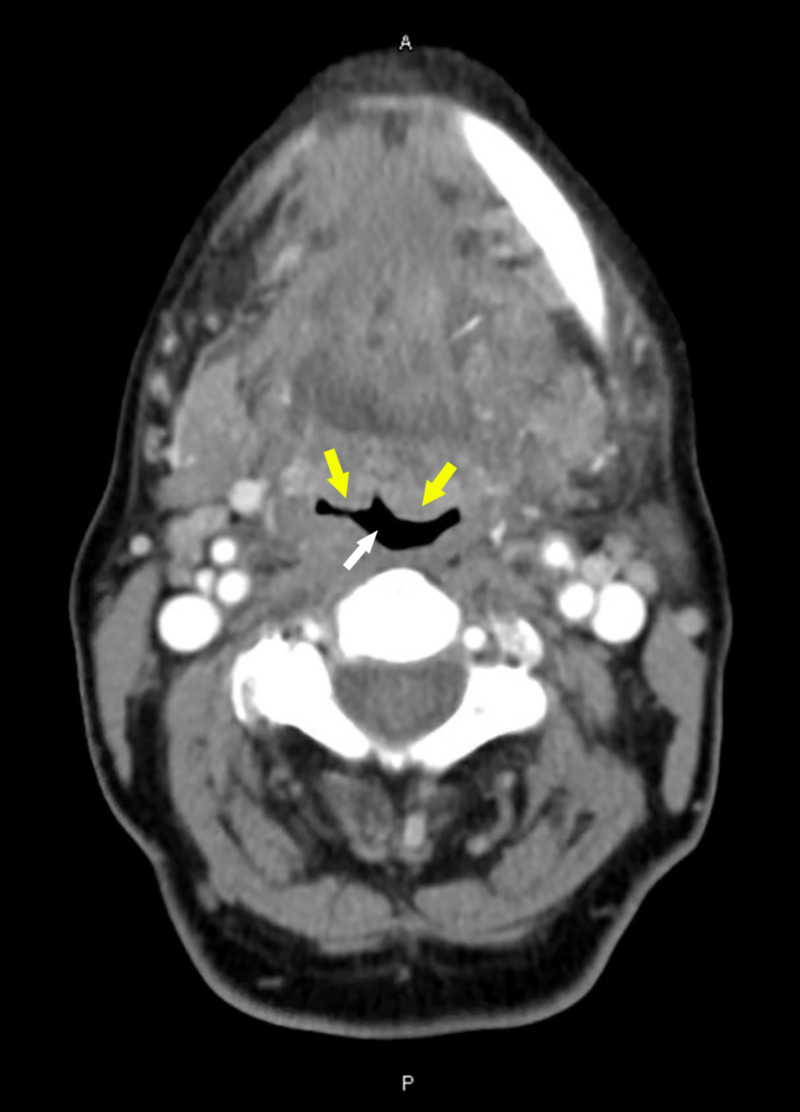
CT of the neck soft tissue revealing LTH (yellow arrows), almost collapsing the airway (white arrow). Only a small crescent-shaped region of the airway remains patent. LTH, lingual tonsillar hypertrophy

**Figure 2:**
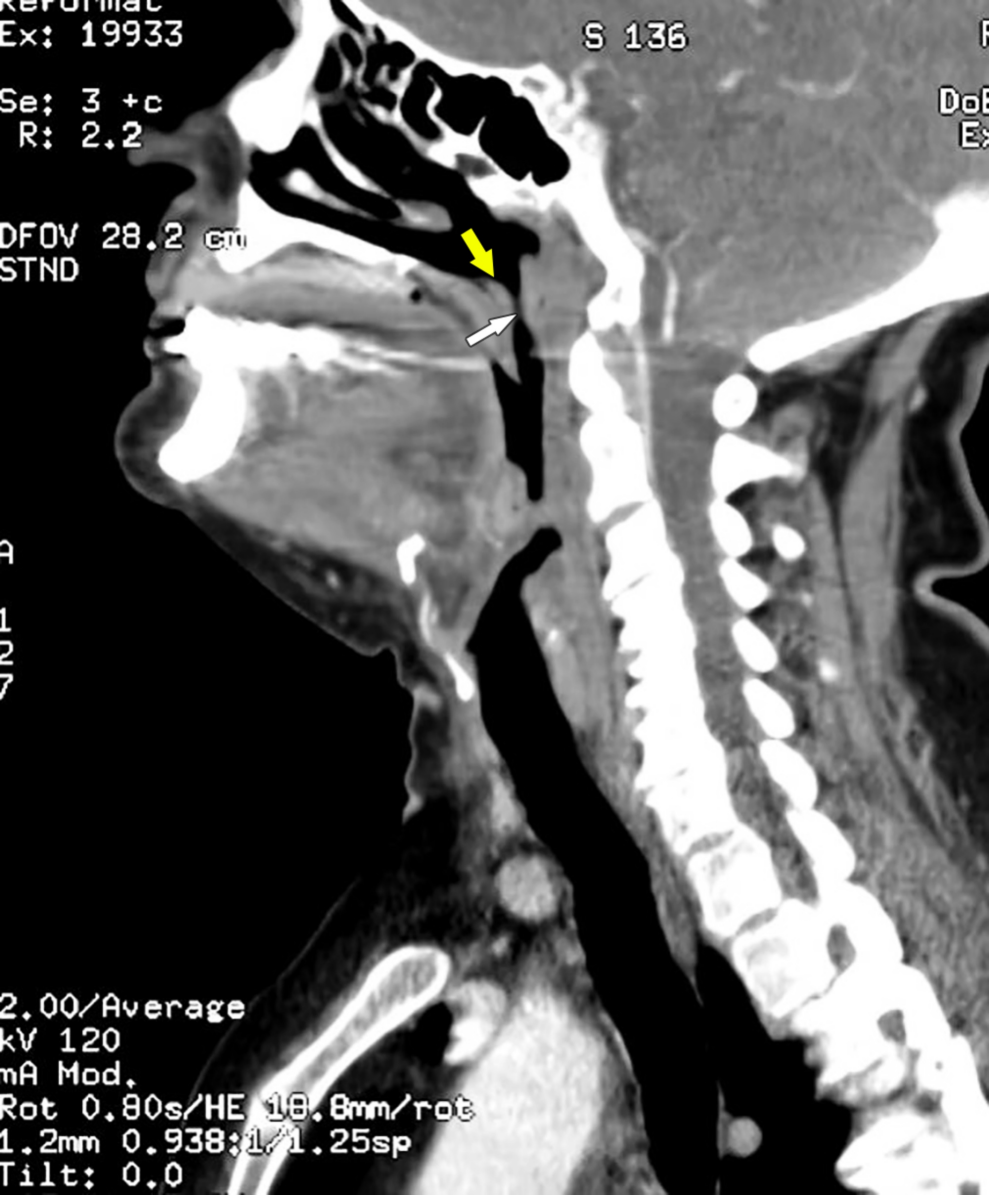
Sagittal view of CT soft tissue neck showing the enlarged lingual tonsils (yellow arrow) partially obstructing the airway (white arrow).

On presentation, the patient’s vital signs were stable: afebrile at 99°F, pulse at 98 beats/min, blood pressure at 160/70 mmHg, respiratory rate at 20 breaths/min, and room air oxygen saturation at 95%. Upon examination, neck fullness with bilateral submandibular tenderness was noted. The patient spoke with a muffled, raspy voice and demonstrated mild dyspnea, but did not have any stridor. On oral examination, no drooling was noted, and the uvula was at the midline. A bedside fiberoptic laryngoscopy was performed under local anesthesia. This revealed an enlarged base of the tongue; bilateral, symmetric vallecular effacement; a thickened epiglottis; and moderate edema of the arytenoids with generalized edema of the larynx, resulting in partial airway obstruction. Laboratory investigation revealed an elevated white blood cell count of 12,300 cells/cu mm (normal range: 4,000-11,000 cells/cu mm) with an elevated neutrophil count at 80% (normal range: 37%-75%). Our initial differential diagnoses included lingual tonsillitis, pharyngitis, and submandibular sialadenitis. A CT scan of the soft tissue of the neck revealed fullness at the base of the tongue, which corresponded to LTH. This, along with laryngeal edema, was the cause of the patient’s partial airway obstruction (Figures 1, 2).

The patient received intravenous (IV) dexamethasone stat to help reduce the airway inflammation and prevent further airway compromise. Broad-spectrum antimicrobial treatment was initiated with IV ampicillin and sulbactam. During her first day of hospitalization, the patient was kept nil per oral and closely observed. The next morning, the patient noted improvement in her symptoms and resolution of her dyspnea. Bedside laryngoscopy revealed reduced laryngeal edema. Anti-reflux therapy with IV pantoprazole was started. Over the next three days, serial laryngoscopies were performed, documenting the steady resolution of the airway obstruction. After the complete resolution of laryngeal edema and airway obstruction, the patient was discharged. She was prescribed an oral antimicrobial course and proton pump inhibitor (PPI) and was placed on an anti-reflux diet. At the follow-up exam a week later, her symptoms were noted to be completely resolved. She was also compliant with her medications and diet.

## Discussion

Waldeyer’s ring, or the nasal-associated lymphoid tissue, is a part of the mucosa-associated lymphoid tissue (MALT) [2-4]. The palatine (faucial), nasopharyngeal (adenoid), and lingual tonsils constitute its major components; the tubal tonsils and lateral pharyngeal bands comprise its minor structures [2-4]. Situated at the entrance of both respiratory and gastrointestinal tracts, this ring-like arrangement of predominantly B-cell-derived lymphoid tissue functions as an immunological gatekeeper, serving as the first line of defense against pathogens [2-4]. As with other lymphoid tissue, tonsillar organs may undergo hypertrophy from continued exposure to allergens or pathogens.

The lingual tonsils are located on the dorsum of the tongue, posterior to the foramen cecum. As such, their hypertrophy (LTH) can displace the epiglottis posteriorly and potentially obstruct the passage of air, liquid, or food through the nasopharynx and oropharynx. This can result in dysphagia as well as airway obstruction [1,3,5]. LTH has been reported in the literature as one of the more unusual causes of unexpected difficulty with airway management, especially in adults [1,5,6]. LTH commonly results from compensatory hypertrophy following palatine tonsillectomy and adenoidectomy. It may also correlate with chronic infections, allergy, gastroesophageal reflux, and obesity [1,5,7]. When associated with allergic rhinitis, for example, LTH is more extensive, covering the entire base of the tongue (grade 3) [8,9].

LTH in adults is mostly asymptomatic. Occasionally, vague symptoms of cough, choking, globus sensation, dysphonia/hoarseness, dysphagia, sore throat, and lingual tonsilloliths may be present [1,5,6,10]. In both children and adults, LTH has been associated with obstructive sleep apnea (OSA) [1,11,12]. LTH has also been implicated in recurrent acute tonsillitis and epiglottitis [1]. LTH has a significant potential for difficult airway management, particularly as it is not evident on routine examination and may lead to fatal airway obstruction [1]. CT evaluation of the soft tissue of the neck is a valuable diagnostic tool in suspected cases [11].

Our patient presented with an uncommon manifestation of LTH that resulted in partial airway obstruction. In a situation that threatens the airway, such as ours, the primary aim is to rapidly restore airway patency. This includes interventions to reduce the airway obstruction and/or to establish an artificial airway while treating the underlying cause. In cases with a compromised airway, the treatment team should be prepared for complete loss of patency. Specialists, like anesthesiologists and intensivists, must be on standby to provide emergency intervention when appropriate. The gold standard technique for non-invasive airway management is fiberoptic bronchoscopy, followed by bag-mask ventilation as managed by an experienced physician. Video laryngoscopy is an alternative solution. In more complex cases, invasive airway management techniques, such as wire-guided retrograde intubation, cricothyroidotomy, and tracheostomy, are performed [5]. Drug treatments for airway obstruction provide particular advantages, especially in regards to halting the progress of inflammation as well as in a prophylactic role. Corticosteroids have demonstrated efficacy in controlling airway inflammation and edema. Though they do have some latency in onset of action, their prophylactic use in preventing post-extubation stridor and reintubation in adults has been widely studied [13]. Corticosteroids act by reducing the inflammatory cellular and cytokine responses; consequently, their application in airway obstruction arising from inflammation and edema has been recommended. Antimicrobials are commonly prescribed to treat any concomitant infections. Preventative therapies limit the triggers for LTH, precluding future hypoxic episodes. Previously undiagnosed gastroesophageal reflux may be managed with a PPI and adherence to an anti-reflux diet to reduce oropharyngeal irritation. If an underlying OSA accompanies LTH, lingual tonsillectomy may be beneficial to open the airway [12].

## Conclusions

LTH is rare and may precipitate airway compromise. Since LTH is not routinely checked during examinations, the definitive treatment of airway obstruction related to it can potentially be delayed. Healthcare providers must regard LTH as a conceivable etiological factor for airway complications and maintain a high degree of clinical suspicion for it whenever airway obstruction occurs. This will pave the way for rapid management, which is crucial for a favorable patient outcome.
